# Enhanced production of gamma-aminobutyrate (GABA) in recombinant *Corynebacterium glutamicum* strains from empty fruit bunch biosugar solution

**DOI:** 10.1186/s12934-018-0977-9

**Published:** 2018-08-21

**Authors:** Kei-Anne Baritugo, Hee Taek Kim, Yokimiko David, Tae Uk Khang, Sung Min Hyun, Kyoung Hee Kang, Ju Hyun Yu, Jong Hyun Choi, Jae Jun Song, Jeong Chan Joo, Si Jae Park

**Affiliations:** 1 Division of Chemical Engineering and Materials Science, Ewha Womans University, 52 Ewhayeodae-gil, Seodaemun-gu, Seoul, 03760 Republic of Korea; 2 Bio-based Chemistry Research Center, Advanced Convergent Chemistry Division, Korea Research Institute of Chemical Technology, P.O. Box 107, 141 Gajeong-ro, Yuseong-gu, Daejeon, 34602 Republic of Korea; 3 Microbial Biotechnology Research Center, Jeonbuk Branch Institute, Korea Research Institute of Bioscience and Biotechnology (KRIBB), 181 Ipsin-gil, Jeongeup, Jeonbuk 56212 Republic of Korea

**Keywords:** *Corynebacterium glutamicum*, Co-utilization, Xylose, Gamma-aminobutyrate, Empty fruit bunch

## Abstract

**Background:**

Recent interest has been focused on the production of platform chemicals from renewable biomass due to increasing concerns on global warming and depletion of fossil fuel reserves. Microbial production of platform chemicals in biorefineries has been suggested to be a promising solution for these problems. Gamma-aminobutyrate (GABA), a versatile bulk chemical used in food and pharmaceutical industry, is also used as a key monomer for nylon 4. GABA can be biologically produced by decarboxylation of glutamate.

**Results:**

In this study, we examined high glutamate-producing *Corynebacterium glutamicum* strains as hosts for enhanced production of GABA from glucose and xylose as carbon sources. An *Escherichia coli gadB* mutant with a broad pH range of activity and *E. coli xylAB* genes were expressed under the control of a synthetic H36 promoter. When empty fruit bunch (EFB) solution was used as carbon source (45 g/L glucose and 5 g/L xylose), 12.54 ± 0.07 g/L GABA was produced by recombinant *C. glutamicum* H36GD1852 expressing *E. coli gadB mutant gene* and *xylAB* genes. Batch fermentation of the same strain resulted in the production of 35.47 g/L of GABA when EFB solution was added to support 90 g/L glucose and 10 g/L xylose.

**Conclusions:**

This is the first report of GABA production by recombinant *C. glutamicum* strains from co-utilization of glucose and xylose from EFB solution. Recombinant *C. glutamicum* strains developed in this study should be useful for an efficient and sustainable production of GABA from lignocellulosic biomasses.

**Electronic supplementary material:**

The online version of this article (10.1186/s12934-018-0977-9) contains supplementary material, which is available to authorized users.

## Background

Biorefinery processes for the production of bio-based chemicals, polymers, and fuels have gained much attention as attractive and practical substitutes for current petroleum-based processes. Among various biorefinery processes, fermentative production of these products from biomass-derived fermentable sugars using microbial host strains is a promising solution for current environmental problems such as global warming and fossil oil depletion, since renewable resources are converted into target products in a carbon-neutral manner [1–6]. In addition, recently developed bioprocesses are now able to utilize a broader range of renewable biomass feedstocks, such as lignocellulosic hydrolysates, algal residue, and recalcitrant coal, for the production of novel chemicals with properties similar or superior to those of conventional petrochemical products [3, 7–19]. Since bio-based polymers from renewable resources are more environmentally friendly and sustainable than petroleum-based polymers, the production of bio-based polymers has been extensively examined to obtain material properties that are same as or superior to those of currently marketed petroleum-based polymers [8, 18–23]. Several bio-based polymers such as polylactic acid (PLA), polybutylene succinate (PBS), and bio-nylons are now commercially available at reasonable market prices [4, 24, 25]. Several diamines, dicarboxylic acids, and amino carboxylic acids can be synthesized in bio-based processes such as microbial fermentation and enzymatic reactions [4]. Nylons synthesized using these bio-based monomers have exhibited excellent material properties, which can be modified by varying carbon numbers and functional groups in monomers. This makes bio-based nylons suitable for a wide range of industrial applications as engineering plastics [2, 24, 25]. Various bio-based nylons such as nylon 4, nylon 510, and nylon 65 have been developed for applications in apparel, food packing, automobile, electronics, and textile industry [4].

Gamma-aminobutyrate (GABA) is a non-protein amino acid that is currently used in the food and pharmaceutical industry as the main component of anti-anxiety drugs, diuretics, and analgesics [26, 27]. Recent application of GABA as a building block chemical in the chemical industry is found in the production of 2-pyrrolidone and nylon 4 [28]. l-Glutamate is the main precursor in biological pathways, in which glutamate is converted into GABA via a single decarboxylation step using glutamate decarboxylase (GAD; EC 4.1.1.15) [27]. Enzymatic conversion of glutamate into GABA has been developed by employing purified glutamate decarboxylase. Moreover, natural and recombinant microorganisms such as lactic acid bacteria [29, 30] and recombinant *E. coli* expressing GAD have been employed for the conversion of glutamate monosodium salt (MSG) into GABA [30–34]. Even though GABA production by direct enzymatic conversion or by whole-cell biotransformation of MSG is efficient, an industrial-scale production of GABA may be highly dependent on environmental conditions such as production location, availability of MSG, and raw material cost [4, 30–34]. Thus, the direct production of GABA from carbon sources such as glucose by fermentation of engineered glutamate-overproducing strains expressing GAD might be practical and cost-effective in a location where biomass-derived sugars are abundant and cheap.

*Corynebacterium glutamicum* is an ideal platform strain for GABA production because it is currently used as a robust industrial microbial cell factory for commercial production of glutamate from glucose [35]. Early efforts to produce GABA using recombinant *C. glutamicum* strain were made through heterologous expression of *Lactobacillus brevis gadBCR* genes encoding glutamate decarboxylase, l-glutamate/GABA antiporter, and transcriptional regulator, which resulted in the production of 2.15 ± 0.16 g/L of GABA with a 160 g/L initial concentration of glucose in the flask culture medium [36]. To enhance GABA production using *C. glutamicum*, deletion of GABA uptake systems was performed to increase intracellular glutamate concentration. Identification, characterization, and deletion of GABA-specific transporter (GabPCg) encoded by *ncgl0464 gene*, together with the expression of *gadB1 gene* from *Lactobacillus brevis*, have increased GABA production in recombinant *C. glutamicum* ATCC 13032 by up to 25.6 ± 2.3 g/L from 50 g/L of glucose [37]. Deletion of the *pknG* gene encoding for serine/threonine protein kinase G and expression of *E. coli* GAD in recombinant *C. glutamicum* enabled the production of 31 g/L of GABA from 100 g/L of glucose [38]. Recently, recombinant *C. glutamicum* ATCC 13032 expressing the *E. coli gadB* mutant gene (Glu89Gln/∆452-466) under the strong synthetic H36 promoter, which encodes a GAD mutant that is active in an expanded pH range of 4–7, was examined for enhanced GABA production from glucose at different pH values (5, 6, 7). This study demonstrated a higher titer of GABA (39 g/L) by a fed-batch culture (pH 6) of a recombinant *C. glutamicum* strain [27].

The cost and availability of feedstock or carbon source are important for the economic feasibility of microbial fermentation. Thus, microbial host strains for fermentative production should be engineered to utilize a wide range of sugars from biomass [4, 39]. Abundant lignocellulosic biomasses are good feedstocks; however, pretreatment and enzymatic hydrolysis are necessary to break down cellulose and hemicellulose components into fermentable sugars, i.e., glucose and xylose [4, 8, 40, 41]. One of the promising raw cellulosic resources for biological production of industrially valuable products is empty fruit bunch (EFB) biosugar solution. It is a solid residue from fresh fruit bunches of oil palm, and is mainly composed of cellulose, hemicellulose, lignin, and ash. Acid hydrolysis, alkali pretreatment, sequential alkali and phosphoric acid treatment, aqueous ammonia and solvent digestion, and hydrothermal treatment with enzymatic hydrolysis of EFB have been developed to obtain sugar solution mainly containing glucose and xylose from EFB [40, 41]. These EFB sugar solutions can be used as carbon sources for microbial production of bio-based fuels such as butanol and ethanol [40].

In this study, we engineered high glutamate-producing *C. glutamicum* strains for co-utilization of glucose and xylose as carbon sources to achieve enhanced production of GABA. Heterologous expression of *E. coli gadB* mutant gene (Glu89Gln/∆452-466) (GAD mutant) and *E. coli xylAB* genes in recombinant *C. glutamicum* ATCC 13032, KCTC 1852, and KCTC 1447 allowed for enhanced production of GABA by co-utilization of glucose and xylose as carbon sources. Furthermore, EFB biosugar solution, which mainly contains glucose with a small amount of xylose, was evaluated as a carbon source for fermentative production of GABA by recombinant *C. glutamicum* H36GD1852.

## Results and discussion

### Examination of glutamate production capacities of *C. glutamicum* strains

**Fig. 1:**
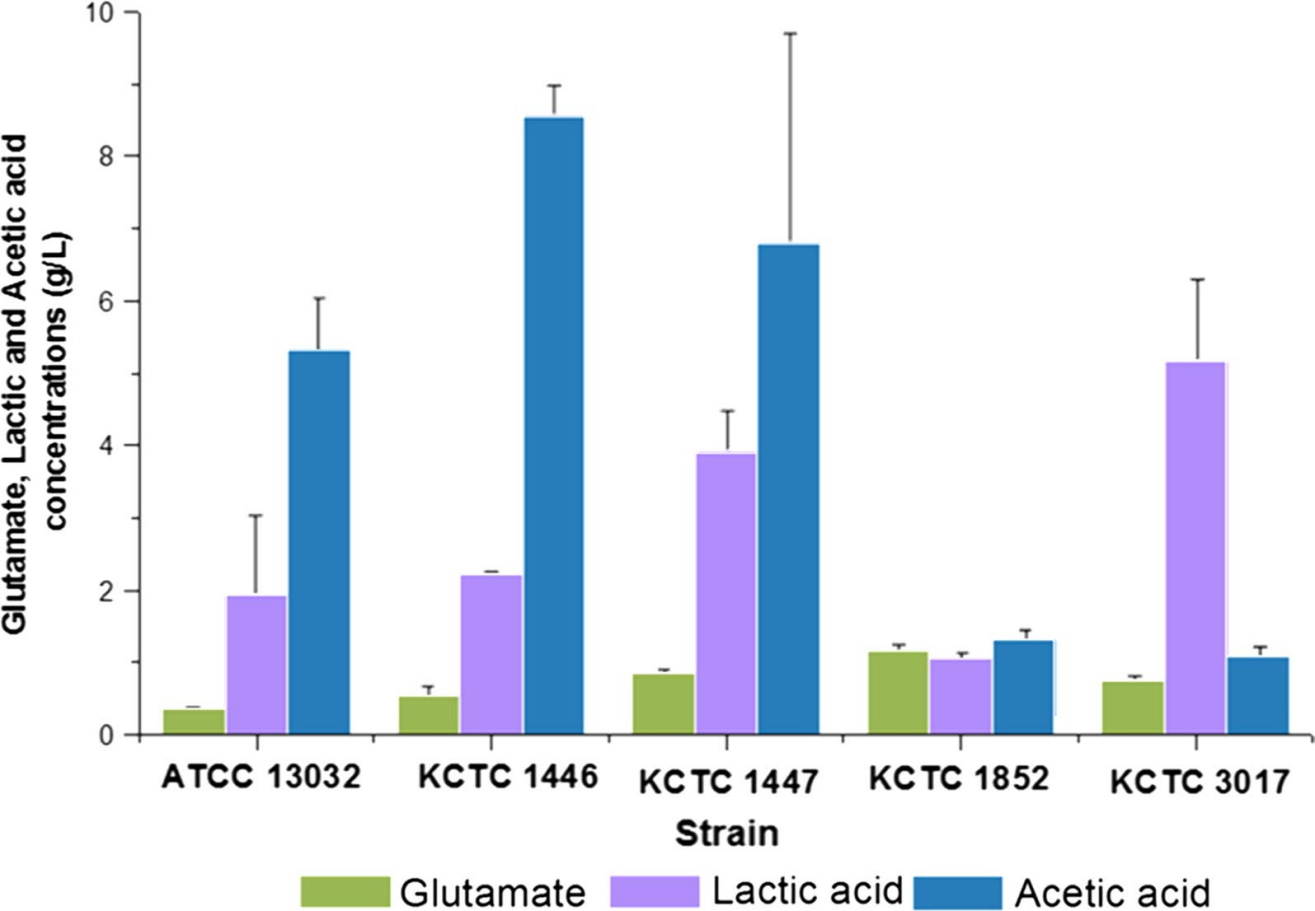
Glutamate production of different *C. glutamicum* strains after 120 h of culture. Concentrations of glutamate, lactic acid, and acetic acid are shown in green, violet, and blue, respectively

We have previously reported the production of GABA by recombinant *C. glutamicum* ATCC 13032 expressing a GAD mutant that was active in an expanded pH range under synthetic promoters capable of varying the strength of protein expression (P_H36_ > P_I16_ > P_L26_) [27]. In this study, glutamate-overproducing *C. glutamicum* strains were cultivated in flask cultures using medium optimized for GABA production. Glutamate production by these strains were evaluated because intracellular accumulation of glutamate will provide more substrate for GAD, leading to enhanced production of GABA. Glutamate over-producing *C. glutamicum* strains were purchased from Korean Collection for Type Cultures (KCTC). *C. glutamicum* KCTC 1446, KCTC 1447, KCTC 1852, and KCTC 3017 were tested along with *C. glutamicum* ATCC 13032 used in a previous study to determine the efficiency of glutamate production among the available strains (Fig. 1). All strains examined in this study produced a higher amount of glutamate compared to a previously used wild-type strain, *C. glutamicum* ATCC 13032 (0.36 ± 0.04 g/L of glutamate). The highest glutamate production was observed in *C. glutamicum* KCTC 1852, which resulted in 1.18 ± 0.06 g/L of glutamate. *C. glutamicum* KCTC 1447, KCTC 3017, and KCTC 1446 produced glutamate to the concentration of 0.86 ± 0.04 g/L, 0.75 ± 0.07 g/L, and 0.54 ± 0.13 g/L, respectively (Fig. 1). The production of usual growth-associated by-products such as lactic acid and acetic acid was also analyzed. The accumulation of by-products indicated that metabolic flux towards the citric acid cycle was reduced, leading to low glutamate production in the *C. glutamicum* strains tested (Fig. 1). Among the strains examined, *C. glutamicum* KCTC 1852, the highest glutamate producer, accumulated a lower concentration of by-products, acetic acid (1.32 ± 0.12 g/L), and lactic acid (1.07 ± 0.05 g/L) compared to other strains. *C. glutamicum* KCTC 1447 showed the second highest titer of glutamate (0.86 ± 0.04 g/L) with an accumulation of lactic acid (3.91 ± 0.54 g/L) and acetic acid (6.80 ± 2.87 g/L). Based on these flask culture results, *C. glutamicum* KCTC1852 and *C. glutamicum* KCTC1447 were determined to have a superior capability for glutamate production. These strains were chosen for further experiments for GABA production despite their accumulation of byproducts such as acetic acid and lactic acid (Fig. 1).

### Development of recombinant *C. glutamicum* strains for GABA production by co-utilization of glucose and xylose

**Fig. 2:**
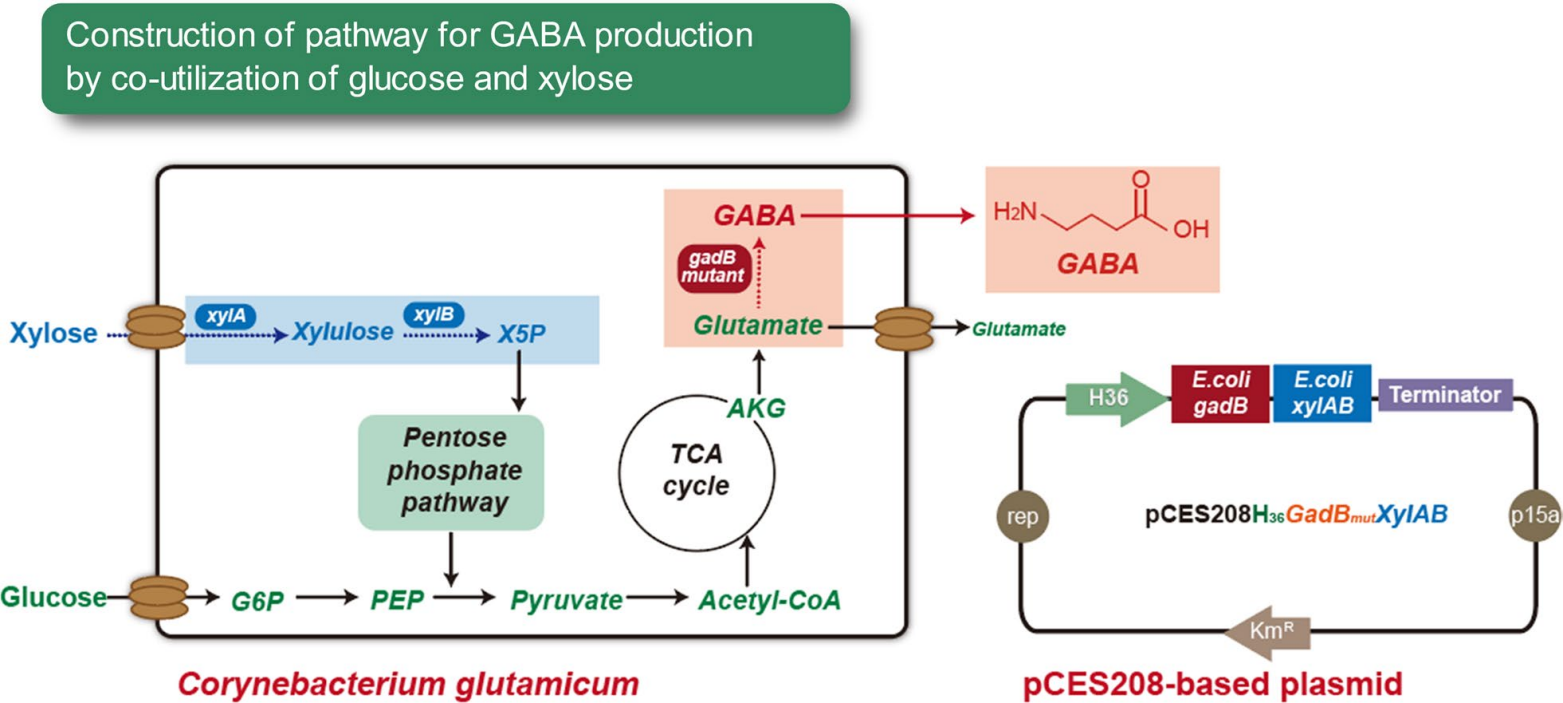
Biosynthetic pathway for the production of gamma-aminobutyrate (GABA) from glucose and xylose as carbon sources using recombinant *C. glutamicum* expressing mutated glutamate decarboxylase and xylose utilization genes from *E. coli*. *G6P* glucose-6-phosphate, *PEP* phosphoenolpyruvic acid, *X5P* xylulose-5-phosphate, *AKG* alpha-ketoglutaric acid

**Fig. 3:**
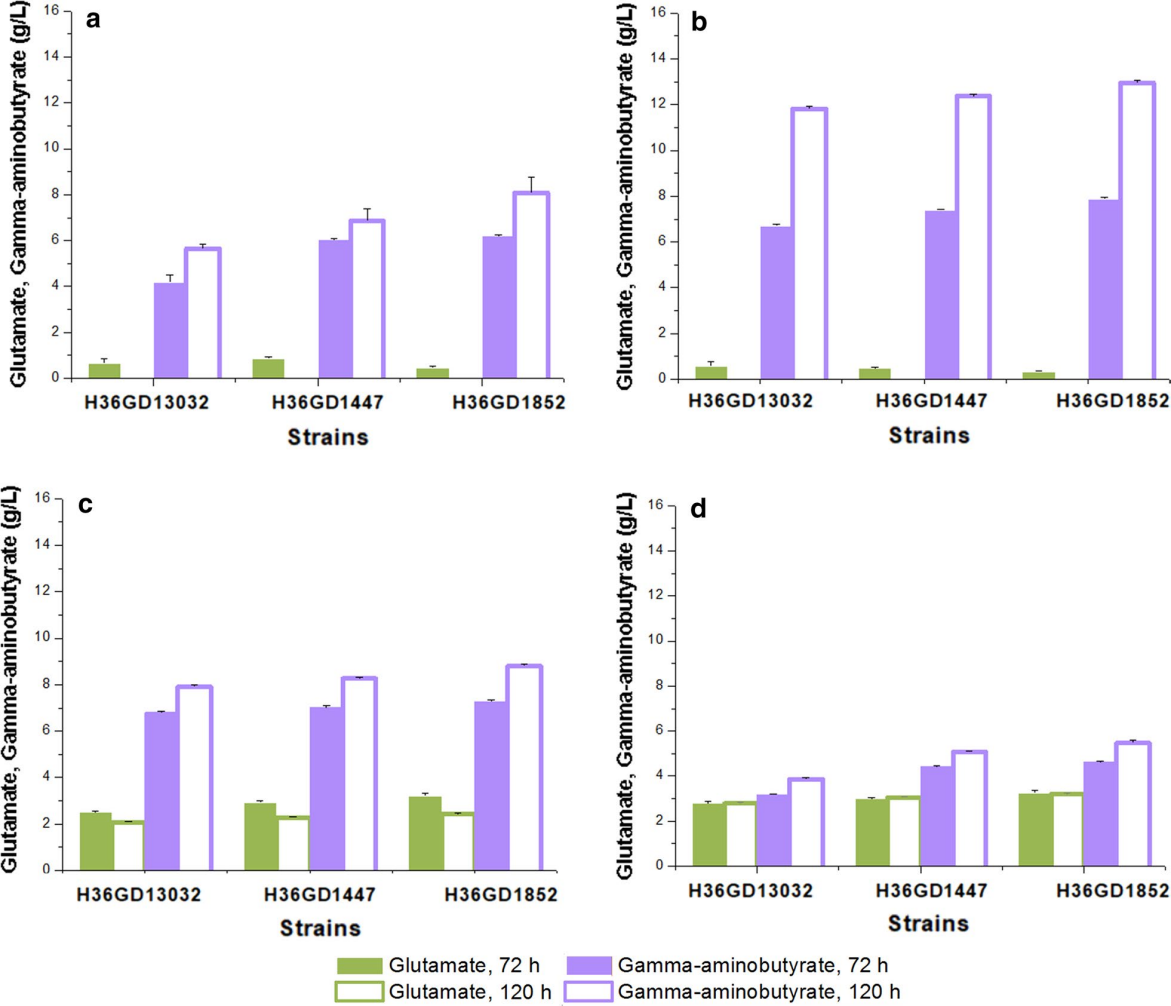
Metabolite production of recombinant *C. glutamicum* H36GD13032, *C. glutamicum* H36GD1447, and *C. glutamicum* H36GD1852 expressing glutamate decarboxylase, xylose isomerase, and xylulose kinase under the strong H36 promoter, after 72 and 120 h of flask cultivation in medium containing different combinations of carbon sources: (**a** 50 g/L glucose; **b** 40 g/L glucose and 10 g/L xylose; **c** 30 g/L glucose and 20 g/L xylose; **d** 20 g/L glucose and 30 g/L xylose. Glutamate, green; Gamma-aminobutyrate, violet; 72 h, solid bar; 120 h, open bar)

To develop recombinant *C. glutamicum* strains for efficient production of GABA by co-utilization of glucose and xylose, *E. coli xylAB* genes and *E. coli* *gadB* mutant gene were introduced into the high glutamate-producing strains, *C. glutamicum* KCTC 1447, KCTC 1852, and control strain *C. glutamicum* ATCC 13032 (Fig. 2). Synthetic promoter-based cassettes were constructed for the expression of the *E. coli* *gadB* mutant gene and *E. coli xylAB* genes encoding xylose isomerase and xylulokinase under the strong promoters P_H30_ and P_H36_ [27, 42]. Six recombinant strains including *C. glutamicum* H30GD1447, H36GD1447, H30GD1852, H36GD1852, H30GD13032, and H36GD13032 were constructed (Table 1). Co-utilization of glucose and xylose for GABA production by the recombinant *C. glutamicum* strains was evaluated by cultivations in different flask cultures containing a total sugar concentration of 50 g/L with various ratios of glucose to xylose (50:0, 40:10, 30:20, and 20:30). Xylose utilization was not observed in *C. glutamicum* H36GM1852 which had no expression of xylose utilization genes from *E. coli* (Additional file 1: Fig. S3). Moreover, GABA production by *C. glutamicum* H36GM1852 was consistently lower compared to *C. glutamicum* H36GD1852 in all culture conditions (Additional file 1: Fig S3). In flask culture with 40:10 ratio of glucose to xylose, *C. glutamicum* H36GD1852 produced more GABA compared to *C. glutamicum* H36GM1852 (12.9 g/L ± 0.09 > 6.7 ± 0.04 g/L). Based on these results, successful xylose utilization was demonstrated in recombinant strains by heterologous expression of xylAB genes (Additional file 1: Fig. S3). There have been few case studies of engineering *C. glutamicum* for the production of lactic acid, succinic acid, 3-hydroxypropionic acid, xylitol and cadaverine by utilization of xylose as carbon source [4, 5]. The production of these biochemicals from xylose were achieved by heterologous expression xylose utilization genes (*xylAB*) from *E. coli* [43, 44]. The ratio of xylose to glucose in the culture medium significantly affected the final concentrations of GABA and glutamate. When a 40:10 ratio of glucose to xylose is present in the medium, the recombinant *C. glutamicum* H36GD13032, H36GD1447, and H36GD1852 strains exhibited the highest production of GABA at 11.83 ± 0.09 g/L, 12.37 ± 0.07 g/L, 12.96 ± 0.09 g/L, respectively (Fig. 3b). The recombinant strains expressing the key genes under strong the P_H30_ promoter showed comparable production of GABA under the same experimental conditions. *C. glutamicum* H30GD13032, H30GD1447, and H30GD1852 strains produced 11.04 ± 0.07, 12.12 ± 0.07 g/L, and 12.73 ± 0.09 g/L of GABA, respectively (Additional file 1: Fig. S2B). When the xylose concentration in the flask culture increased from 10 to 30 g/L, all recombinant strains produced less GABA while more glutamate was detected in the medium. The production of GABA by the recombinant *C. glutamicum* H36GD1852 strain decreased from 12.96 ± 0.09 to 5.46 ± 0.12 g/L, but glutamate increased from 0 to 3.21 ± 0.05 g/L (Fig. 3). When key genes were expressed under the strong P_H30_ promoter in *C. glutamicum* H30GD1852, flask cultivation with 40:10 and 30:20 glucose-to-xylose ratios produced 12.73 ± 0.09 and 5.31 ± 0.12 g/L of GABA, respectively, while glutamate accumulation increased from 0 to 3.00 ± 0.07 g/L. The highest concentration of GABA (12.96 ± 0.09 g/L) was obtained by the recombinant *C. glutamicum* H36GD1852 strain in flask culture with 40 g/L of glucose and 10 g/L of xylose. This value was 60.4% higher than GABA production from 50 g/L of glucose (8.01 ± 0.67 g/L) by the same strain, and two-fold higher GABA production from 50 g/L of glucose (6.32 ± 0.38 g/L) by recombinant *C. glutamicum* ATCC 13032 expressing the *E. coli* *gadB* mutant gene in our previous study [27].

**Table 1:** Strains and plasmids used in this study

Strains and plasmids	Relevant characteristics	References or sources
Strains		
*E. coli* XL1-Blue	*recA1 endA1 gyrA96 thi*-*1 hsdR17 supE44 relA1 lac* [FA1*proAB lacI*^*q*^*ZΔM15* Tn*10* (Tet^R^)]	Stratagene
*C. glutamicum* strains KCTC 1446, 1447, 1852, 3017	Glutamate producers	KCTC
*C. glutamicum* ATCC 13032	Wild type	ATCC
*C. glutamicum* H30GD13032	*C. glutamicum* ATCC 13032 harboring pCES208H30 GadBmutXylAB	This study
*C. glutamicum* H36GD13032	*C. glutamicum* ATCC 13032 harboring pCES208H36 GadBmutXylAB	This study
*C. glutamicum* H30GD1447	*C. glutamicum* KCTC 1447 harboring pCES208H30 GadBmutXylAB	This study
*C. glutamicum* H36GD1447	*C. glutamicum* KCTC 1447 harboring pCES208H36 GadBmutXylAB	This study
*C. glutamicum* H30GD1852	*C. glutamicum* KCTC 1852 harboring pCES208H30 GadBmutXylAB	This study
*C. glutamicum* H36GD1852	*C. glutamicum* KCTC 1852 harboring pCES208H36 GadBmutXylAB	This study
*C. glutamicum* H36GM1852	*C. glutamicum* KCTC 1852 harboring pCES208H36 GadBmut	This study
Plasmids		
pCES208H30GFP	pCES208 derivative; P_H30_, eGFP, Km^r^	[42]
pCES208H30GFP	pCES208 derivative; P_H36_, eGFP, Km^r^	[42]
pHGmut	pCES208 derivative; P_H36_, *gadB* mutant gene from *E. coli* (Glu89Gln/Δ452-466), Km^r^	[27]
pCES208H30 GadBmutXylAB	pCES208H30GFP derivative; P_H30_, *gadB* mutant gene from *E. coli* (Glu89Gln/Δ452-466), xylose utilization genes *xylAB* from *E. coli*, Km^r^	This study
pCES208H36 GadBmutXylAB	pHGmut derivative; P_H36_, *gadB* mutant gene from *E. coli* (Glu89Gln/Δ452-466) xylose utilization genes *xylAB* from *E. coli*, Km^r^	This study

The enhanced production of GABA due to the co-utilization of glucose and xylose is dependent on the amount of xylose in the medium. In a previous report, adjusting the ratio of glucose to xylose in the culture medium improved the production of succinic acid by recombinant *E. coli* strain. It was concluded that the production of succinic acid was regulated by the ratio of glucose and xylose [45]. In this study, similar results were observed. Highest production of GABA was obtained in the presence of 10 g/L of xylose (Fig. 3b). However, a significant decrease in GABA production was observed for all recombinant strains when the concentration of xylose exceeded 10 g/L in flask cultivation, i.e., glucose-to-xylose ratios of 30:20 and 20:30 (Fig. 3c, d). Simultaneous uptake of glucose and xylose was observed for all mixed culture setups. The complete consumption of glucose was achieved after 72 h of cultivation (Additional file 1: Fig. S1). However, xylose utilization took up to 120 h when the concentration of xylose exceeded 10 g/L (Additional file 1: Fig. S1C, D). Slow xylose consumption, increased accumulation of glutamate and decreased production of GABA was also observed as the ratio of xylose in the mixed culture increased (Additional file 1: Fig. S1C, D; Fig. 3c, d). To investigate if this observation is due to the increased concentration of xylose in culture medium, the best GABA producing strain, H36GD1852 was cultivated culture medium with different concentrations of xylose (5–30 g/L) and fixed concentration of glucose (20 g/L) (Additional file 1: Fig. S4). The recombinant strain H36GD1852 produced higher concentration of GABA from 30 g/L of xylose and 20 g/L of glucose compared to when only 20 g/L of glucose was used as carbon source in culture medium (7.71 ± 0.36 g/L > 3.33 ± 0.26 g/L). GABA production in flask cultures with glucose-to-xylose ratio of 40:10 (12.37 ± 0.07 g/L) and 50:0 (8.01 ± 0.67 g/L) still produced the higher titer of GABA compared to the data obtained from the additional flask culture with 20:30 glucose-to-xylose ratio (7.71 g/L) (Fig. 3a, b; Additional file 1: Fig. S4). However, glutamate accumulation of 2–3 g/L was still observed when concentration of xylose in culture medium exceeded 10 g/L (Additional file 1: Fig. S4). Complete xylose utilization was observed for all culture conditions except when 30 g/L of xylose was in culture medium. After 120 h of cultivation, 1 g/L of xylose was still detected in culture medium (data now shown).

In the presence of a higher ration of xylose in the culture medium, the increase in glutamate accumulation and decrease in GABA production may be attributed to a decreased in the intracellular ATP pool for biosynthesis of pyridoxal 5′-phosphate (PLP) because ATP is also required for the conversion of xylulose to xylulose 5-phosphate [45, 46]. GAD requires PLP as cofactor for the conversion of glutamate into GABA and GAD activity was affected by the lower intracellular PLP pool, resulting in decreased GABA production and increased glutamate accumulation proportional to the xylose concentration. To investigate the role of PLP in GABA production *C. glutamicum* H36GD1852 was cultivated with 30:20 glucose/xylose ratio as carbon source and different concentrations of PLP from 0.1 to 0.4 mM. In the presence of 0.1 mM of PLP, 3.5 ± 0.47 g/L of glutamate accumulation and 5.2 ± 0.08 g/L of GABA production was observed (Fig. 3b, c). When PLP concentration was increased from 0.1 to 0.4 mM, accumulation of glutamate remarkably decreased from 3.5 ± 0.47 g/L to 0.48 ± 0.22 g/L and GABA production increased from 5.2 ± 0.08 to 7.6 ± 0.26 g/L of GABA (Additional file 1: Fig. S5). The decreased glutamate accumulation and increased GABA production observed here is the direct effect of adding increasing amounts of PLP during flask cultivation. Based on these results, further metabolic redesign for enhanced xylose utilization and PLP production would be the next step in increasing the production of GABA from xylose.

### Co-utilization of glucose and xylose in EFB biosugar solution for GABA production by recombinant *C. glutamicum* H36GDX1852

**Fig. 4:**
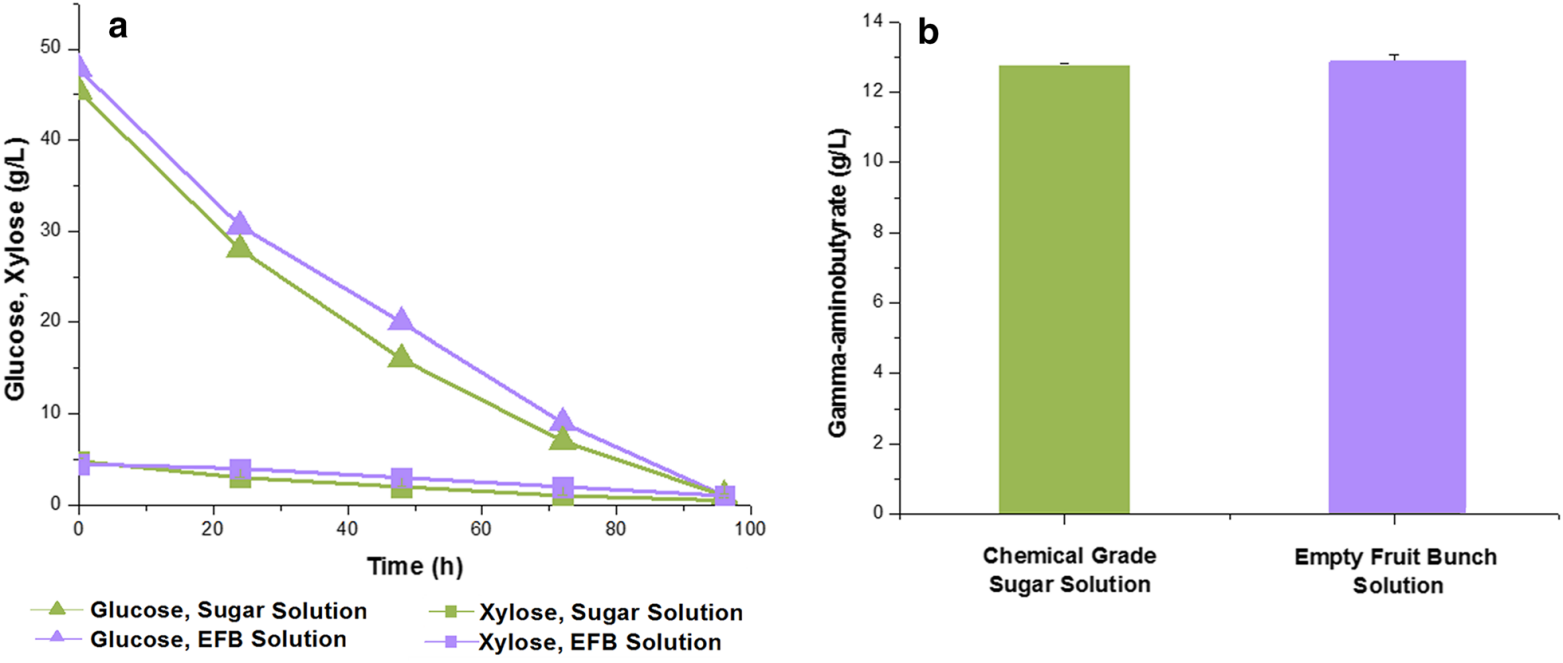
Time profile of carbon utilization (**a**) and gamma-aminobutyrate production (**b**) of recombinant *C. glutamicum* KCTC H36GD1852 expressing glutamate decarboxylase, xylose isomerase, and xylulose kinase under the strong H36 promoter in a shake flask culture set-up with empty fruit bunch (EFB) biosugar solution and chemical-grade sugars with different ratios of glucose (45 g/L) to xylose (5 g/L) after 120 h. EFB Solution (glucose, purple filled up triangle; xylose, purple filled square); Chemical-grade sugar solution (glucose, green filled up triangle; xylose, green filled square)

The recombinant *C. glutamicum* H36GD1852 strain was further examined for whether the carbon co-utilization system developed in this study can be efficiently used in the lignocellulosic bio-refinery processes. EFB biosugar, a biomass-derived mixed glucose-xylose solution developed in our previous study [41], was used as the carbon source for GABA production. A chemical-grade sugar mixture was used as the carbon source for control study. After pretreatment of EFB, xylan in hemicellulose was removed as part of the liquid fraction, and glucose in the glucan fraction was the predominantly species in the developed biosugar system. The original EFB solution used in this study was composed of 38.3% glucose, 2.92% xylose, 0.48% acetic acid, 0.03% protein, and 0.61% phenolics (Additional file 1: Table S1). The final concentrations of glucose and xylose in the EFB solution after sterilization were 284 and 24.5 g/L, respectively. A total of 50 g/L of sugars from sterilized EFB solution was added in the flask culture, and the initial concentrations of glucose and xylose were detected as 45 and 5 g/L, respectively. In the flask culture with EFB solution, the *C. glutamicum* H36GD1852 strain could completely consume glucose and xylose after 96 and 120 h, respectively (Fig. 4a). There was no significant difference in the rate of carbon utilization between chemical-grade glucose/xylose and EFB solution as the carbon source for the recombinant *C. glutamicum* H36GD1852 strain despite the presence of low concentrations of acetic acid (0.48%) or phenolics (0.61%) in the EFB solution (Fig. 4a and Additional file 1: Table S1). In addition, GABA production (12.54 ± 0.07 g/L) from the EFB solution was comparable to GABA concentration (12.79 ± 0.04 g/L) obtained from chemical-grade glucose and xylose (Fig. 4b).

### Fermentations of recombinant *C. glutamicum* H36GD1852 for the production of GABA using glucose and xylose from the EFB biosugar solution

Batch fermentations were performed to investigate the efficiency of recombinant *C. glutamicum* H36GD1852 in the co-utilization of glucose and xylose in the EFB solution. Batch fermentations were carried out at pH 6 and 7 because the GAD employed in this study exhibited high production of GABA at this pH condition [27].

**Fig. 5:**
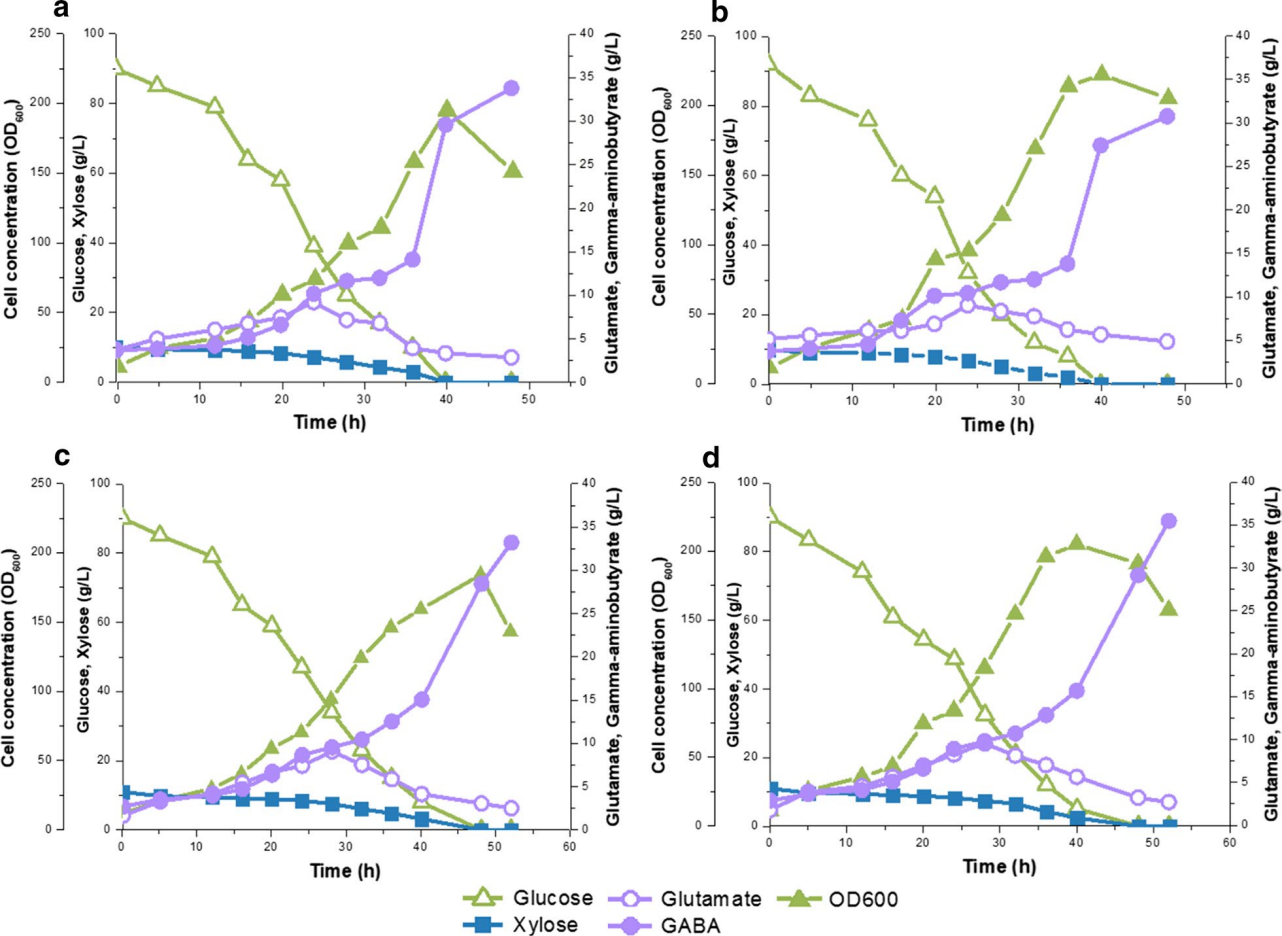
Time profile of carbon source utilization and metabolite production of recombinant *C. glutamicum* H36GD1852 expressing the key genes encoding glutamate decarboxylase, xylose isomerase, and xylulokinase from *E. coli* under the strong synthetic promoter H36 in batch culture fermentation using chemical-grade sugars at pH 6 (**a**) and pH 7 (**b**), and empty fruit bunch biosugar at pH 6 (**c**) and pH 7 (**d**) with a glucose-to-xylose ratio of 90:10. Cell growth, green filled triangle; glucose, green open triangle; xylose, blue filled square; glutamate, purple open circle; GABA, purple filled circle

As shown in Fig. 5b, d, comparable cell growth was observed in the EFB solution (OD_600_ 205) and chemical-grade glucose and xylose medium (OD_600_ 214) at pH 7 after a 40-h cultivation. However, the cell density (OD_600_ 159, Fig. 5c) was slightly lower in the EFB solution at pH 6 after a 40-h cultivation compared to that (OD_600_ 195, Fig. 5a) obtained in the chemical-grade glucose and xylose medium after 40 h. The optimal pH for *C. glutamicum* H36GD1852 growth was pH 7 [27], and thus slightly lower growth was observed at pH 6. The EFB solution initially contained fermentation inhibitors, 0.48% acetic acid, and 0.61% phenolics (Additional file 1: Table S1). However, no significant cell growth inhibition was observed in the EFB solution at pH 7 even though *C. glutamicum* cells are known to be more susceptible to growth retardation by fermentation inhibitors when grown in aerobic conditions compared to growth-inhibited cells under anaerobic conditions [47, 48].

Similar concentrations of GABA (30.76–35.47 g/L) were obtained in batch fermentations with chemical-grade sugars and EFB biosugar containing 90 g/L glucose and 10 g/L xylose at pH 6 and 7 (Fig. 5b, d). The highest concentration of GABA (35.47 g/L) was produced by recombinant *C. glutamicum* H36GD1852 in the EFB solution at pH 7 (Fig. 5d). Furthermore, 33.21 g/L of GABA was produced in the batch fermentation with the EFB solution at pH 6, which was comparable to the 33.79 g/L of GABA obtained from chemical-grade sugars at pH 6 (Fig. 5a, c). These results demonstrated that the EFB solution can be used as a good carbon source, with GABA production yields that were comparable to that obtained from chemical-grade glucose and xylose. When mixed sugars were used as a carbon source during fermentation, diauxic growth was typically observed due to catabolite repression. Glucose was utilized first before other available carbon sources such as xylose or arabinose [48]. However, in our study, a typical pattern of carbon catabolite repression was not observed when the recombinant strains were cultivated by batch fermentation using the EFB solution. Both glucose and xylose uptakes were observed at the start of fermentation (Fig. 5). A similar trend was observed when recombinant *C. glutamicum* CRX2 and *C. glutamicum* CgEcXylBA strain expressing xylose isomerase and xylulokinase genes from *E. coli* was used for the co-utilization of glucose and xylose at a 2:1 ratio [43, 44]. The recombinant *C. glutamicum* strains developed in this study can be further engineered for GABA production in consolidated bioprocessing using other hemicellulosic carbon sources, such as xylan, by establishing pathways for enhanced xylan degradation, xylose utilization, and transport [4]. One of the promising raw cellulosic resources for bio-based production of industrially valuable products is EFB biosugar solution. Further investigations are required to identify critical factors affecting the cell growth, glucose and xylose co-utilization, and GABA production from the EFB solution.

## Conclusions

This study is the first report of engineering *C. glutamicum* for enhanced production of GABA by co-utilization of glucose and xylose in the EFB solution as the carbon source. No significant carbon catabolite repression was observed in the culture medium with increased concentrations of xylose. Batch fermentation of the engineered *C. glutamicum* H36GD1852 strain resulted in the production of 35.47 g/L GABA from 90 g/L glucose and 10 g/L xylose in the EFB solution. *C. glutamicum* engineered to utilize both xylose and glucose developed in this study could be useful for efficient and sustainable GABA production from lignocellulosic biomass.

## Methods

### Bacterial strains and plasmids

All bacterial strains and plasmids used in this study are listed in Table 1. *E. coli* XL1-Blue (Stratagene, La Jolla, CA, USA) was used for general gene cloning studies. *C. glutamicum* KCTC 1447 and 1852 strains were purchased from the Korean Collection for Type Cultures (KCTC, Korea). The plasmids pCES208H30GFP/pCES208H36GFP [42] and pHGmut containing the *E. coli gadB* mutant gene (Glu89Gln/Δ452-466 gene) [27] were constructed as previously described.

### Plasmid construction

All DNA manipulations were performed following standard procedures [49]. Polymerase chain reaction (PCR) was performed with the C1000 Thermal Cycler (Bio-Rad, Hercules, CA, USA). Primers used in this study were synthesized at Bioneer (Daejeon, Korea). The GAD enzyme used in this study is a mutated version of *E. coli* GAD used in our previous study. It has a mutation (Glu89Gln/Δ452-466) which makes it active in expanded pH range of up to pH 7, which is optimum pH of C. *glutamicum* cell growth [27]. The plasmids pCES208H30GFP and pCES208H36GFP, were used as backbone plasmid for construction of pCES208H30GadBmutXylAB and pCES208H36GadBmutXylAB [42]. The plasmid pHGmut containing *E. coli gadB* mutant gene (Glu89Gln/Δ452-466) was used as source of mutated *gadB* gene and was constructed as previously described [27]. The plasmid pCES208H30GadBmut was constructed by replacing the green fluorescent protein (GFP) gene of plasmid pCES208H30GFP with the *E. coli gadB* mutant gene obtained from pHGmut via digestion with *Bam*HI and *Not*I [27]. The *E. coli xylAB* genes were amplified from *E. coli* XL1-Blue chromosomal DNA using primers 1 (**gcggccgc**atgcaagcctattttgaccagc) and 2 (**gcggccgc**ttacgccattaatggcagaag), and then inserted into pCES208H30GadBmut at the *Not*I site to produce pCES208H30GadBmutXylAB. The plasmid pCES208H36GadBmutXylAB was constructed in the same manner, in which the only difference was that pHGmut was used as the backbone plasmid for insertion of the *E. coli xylAB* genes.

### Culture conditions

*Escherichia coli* XL1-Blue, used for general gene cloning experiments, was cultured at 37 °C in Luria–Bertani (LB) medium (10 g/L tryptone, 5 g/L yeast extract, and 5 g/L NaCl). Flask cultures of the wild-type and recombinant strains of *C. glutamicum* were carried out in triplicate at 30 °C and 250 rpm in a rotary shaker. The GP1 medium optimized in our previous study [27] was used for screening of glutamate and GABA-producing strains. The GP1 medium for flask cultivation contained (per liter): 50 g of (NH_4_)_2_SO_4_, 1 g of K_2_HPO_4_, 3 g of urea, 0.4 g of MgSO_4_·7H_2_O, 50 g of peptone, 0.01 g of FeSO_4_, 0.01 g of MnSO_4_·5H_2_O, 200 μg of thiamine, and 1 mM of pyridoxal 5′-phosphate hydrate (PLP) [27]. PLP was added to culture medium because it is a cofactor of glutamate decarboxylase. 0.1 mM of PLP was the best concentration of PLP for prolonging GABA production using recombinant *C. glutamicum* strains [46]. Kanamycin and biotin was added to the culture medium at 25 and 50 μg/L, respectively. Only 50 μg/L of biotin was used in flask culture because biotin-limited conditions promotes glutamate production [27]. CaCO_3_ was added to the culture medium at 10 g/L to minimize the pH change during cultivation. Glucose and xylose were used as carbon sources at different concentrations (5–40 g/L). The EFB solution used as carbon source was prepared as described previously [41]. The glucose, xylose and EFB solutions used in this study were autoclaved at 121 °C for 15 min. EFB solution was filtered to remove small particles prior to autoclave sterilization. The composition of EFB solution is presented in Additional file 1: Table S1.

For preparation of the seed culture for batch fermentation, 4 mL of overnight culture of recombinant *C. glutamicum* was used to inoculate 50 mL of GP1 medium in a 500 mL baffled flask. Batch fermentations were carried out at 30 °C and 600 rpm in a 2.5-L jar fermentor (BioCNS, Korea) containing 500 mL of CG100 medium composed of (per liter) 90 g of glucose, 10 g of xylose, 30 g of yeast extract, 30 g of (NH_4_)_2_SO_4_·7H_2_O, 0.5 g of KH_2_PO_4_, 0.5 g of MgSO_4_·7H_2_O, 0.01 g of MnSO_4_·H_2_O, 0.01 g of FeSO_4_·7H_2_O, 0.5 mg of biotin, and 0.3 mg of thiamine-HCl. Kanamycin was added to the culture medium at 25 µg/mL [50]. Two different pH values (6.0 and 7.0) were examined for GABA production and controlled by automatic addition of 28% (v/v) NH_4_OH. Foam formation was suppressed by adding Antifoam 204 (Sigma-Aldrich, St. Louis, MO, USA), and cell growth was monitored by measuring the optical density at 600 nm (OD_600_) with UV–Visible spectrophotometer (UV-PharmaSpec 1700, Shimadzu).

The EFB solution was filtered using a 0.22-µm membrane before autoclave sterilization at 121 °C for 15 min. Sterilized EFB solution contained 284.39 g/L glucose and 24.51 g/L xylose. For the flask culture and batch fermentations using EFB solution as the carbon source, the total sugar concentrations were maintained at 50 and 100 g/L, respectively. The final concentration of sugars when the EFB solution was used in the flask culture were 45 g/L glucose and 5 g/L xylose. For batch fermentations using the EFB solution, the final concentration of glucose and xylose were 90 g/L and 10 g/L, respectively. Batch fermentation using chemical-grade sugars with the same ratio of glucose to xylose was included as the control experiment for comparison of growth and GABA production.

### Analysis

Concentrations of glucose, xylose, and organic acids were determined by high performance liquid chromatography (HPLC). Concentrations of GABA and glutamate were determined by HPLC using Optimapak C18 column (RStech, DaeJeon, Korea) as previously reported [51].

## Additional file

**Additional file 1: Table S1.** Composition of empty fruit bunch (EFB) solution used as the carbon source. **Figure S1.** Time profiles of carbon utilization of recombinant *C. glutamicum* strains H36GD13032 (○, ○), H36GD1447 (△, △), and H36GD1852 (□, □) using different combinations of carbon sources. Glucose consumption is indicated as green lines and xylose is represented as blue lines (A: 50 g/L glucose, B: 40 g/L glucose, 10 g/L xylose, C: 30 g/L glucose and 20 g/L xylose, D: 20 g/L glucose and 30 g/L xylose). **Figure S2.** Gamma-aminobutyrate production by recombinant *C. glutamicum* strains H30GD13032, H30GD1447, and H30GD1852 after 120 h of flask cultivation in medium containing different combinations of carbon sources (A, 50 g/L glucose; B, 40 g/L glucose and 10 g/L xylose; C, 30 g/L glucose and 20 g/L xylose; D, 20 g/L glucose and 30 g/L xylose). **Figure S3.** Concentrations of xylose, glutamate and gamma-aminobutyrate after 120 h of flask cultivation using recombinant *C. glutamicum* H36GM1852 (gray) and *C. glutamicum* H36GD1852 (white). The culture medium used contained different combinations of carbon sources (50G, 50 g/L glucose; 40G10X, 40 g/L glucose and 10 g/L xylose; 30G20X, 30 g/L glucose and 20 g/L xylose; 20G30X, 20 g/L glucose and 30 g/L xylose). **Figure S4.** Gamma-aminobutyrate production by recombinant *C. glutamicum* H36GD1852 after 120 h of flask cultivation in medium containing different combinations of carbon sources (50G, 50 g/L glucose; 20G, 20 g/L glucose; 20G5X, 20 g/L glucose and 50 g/L xylose; 20G10X, 20 g/L glucose and 10 g/L xylose; 20G20X, 20 g/L glucose and 20 g/L xylose; 20G3X, 20 g/L glucose and 30 g/L xylose). **Figure S5.** Gamma-aminobutyrate production and glutamate accumulation by recombinant *C. glutamicum* H36GD1852 after 120 h of flask cultivation in medium containing 30:20 glucose to xylose ratio. Additional concentrations of PLP (0.1–0.4mM) was supplemented during cultivation.
