# Supplementary material for: Enhanced production of gamma-aminobutyrate (GABA) in recombinant Corynebacterium glutamicum strains from empty fruit bunch biosugar solution
Source: Microb Cell Fact. 2018 Aug 21;17:129. doi: 10.1186/s12934-018-0977-9 (PMC6102818; doi:10.1186/s12934-018-0977-9)

**Additional Files:**

Table S1. Composition of empty fruit bunch solution used as carbon source Data presented are means ± standard deviations.

| Component | Composition  (g/g, %) |
| --- | --- |
| **Glucose** | 38.3 ± 0.00 |
| **Xylose** | 2.92 ± 0.00 |
| **Acetic acid** | 0.48 ± 0.00 |
| **Protein** | 0.03 ± 0.00 |
| **Phenolics** | 0.31 ± 0.02 |

Figure S1. Time profiles of carbon utilization of recombinant *C. glutamicum* strains H36GD13032 (○, ○), H36GD1447 (△, △), and H36GD1852 (□, □) using different combinations of carbon sources. Glucose consumption is indicated as green lines and xylose is represented as blue lines. (A: 50 g/L glucose, B: 40 g/L glucose, 10 g/L xylose, C: 30 g/L glucose and 20 g/L xylose, D: 20 g/L glucose and 30 g/L xylose).


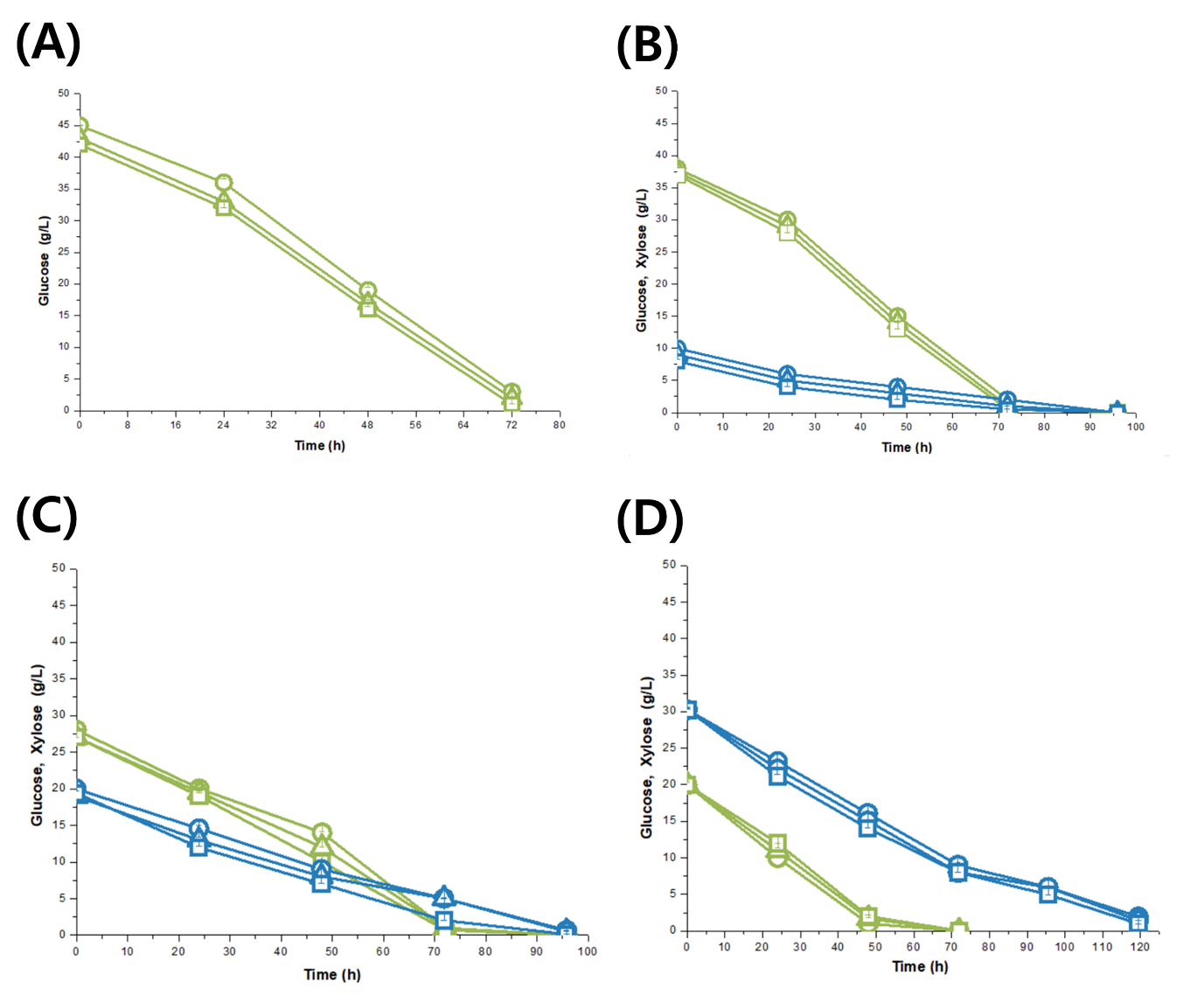


Figure S2. GABA production by recombinant *C. glutamicum* strains H30GD13032, H30GD1447, and H30GD1852 after 120 h of flask cultivation in medium containing different combinations of carbon sources (A: 50 g/L glucose, B: 40g/L glucose and 10 g/L xylose, C: 30 g/L glucose and 20 g/L xylose, D: 20 g/L glucose and 30 g/L xylose).


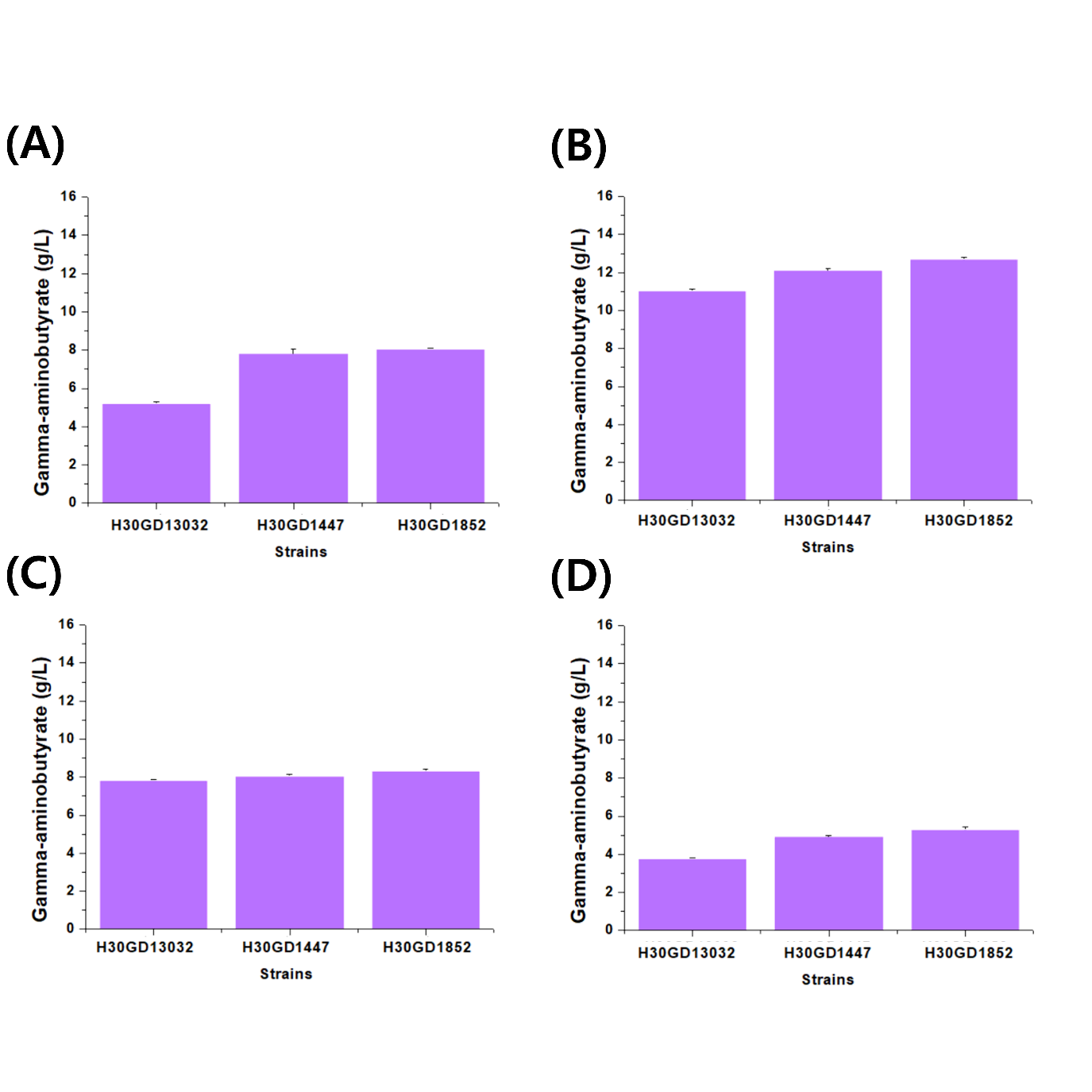


Figure S3. Concentrations of xylose, glutamate and gamma-aminobutyrate after 120H of flask cultivation using recombinant *C.glutamicum* H36GM1852 (gray) and *C. glutamicum* H36GD1852 (white). The culture medium used contained different combinations of carbon sources (50G, 50 g/L glucose; 40G10X, 40 g/L glucose and 10 g/L xylose; 30G20X, 30 g/L glucose and 20 g/L xylose; 20G30X, 20 g/L glucose and 30 g/L xylose).


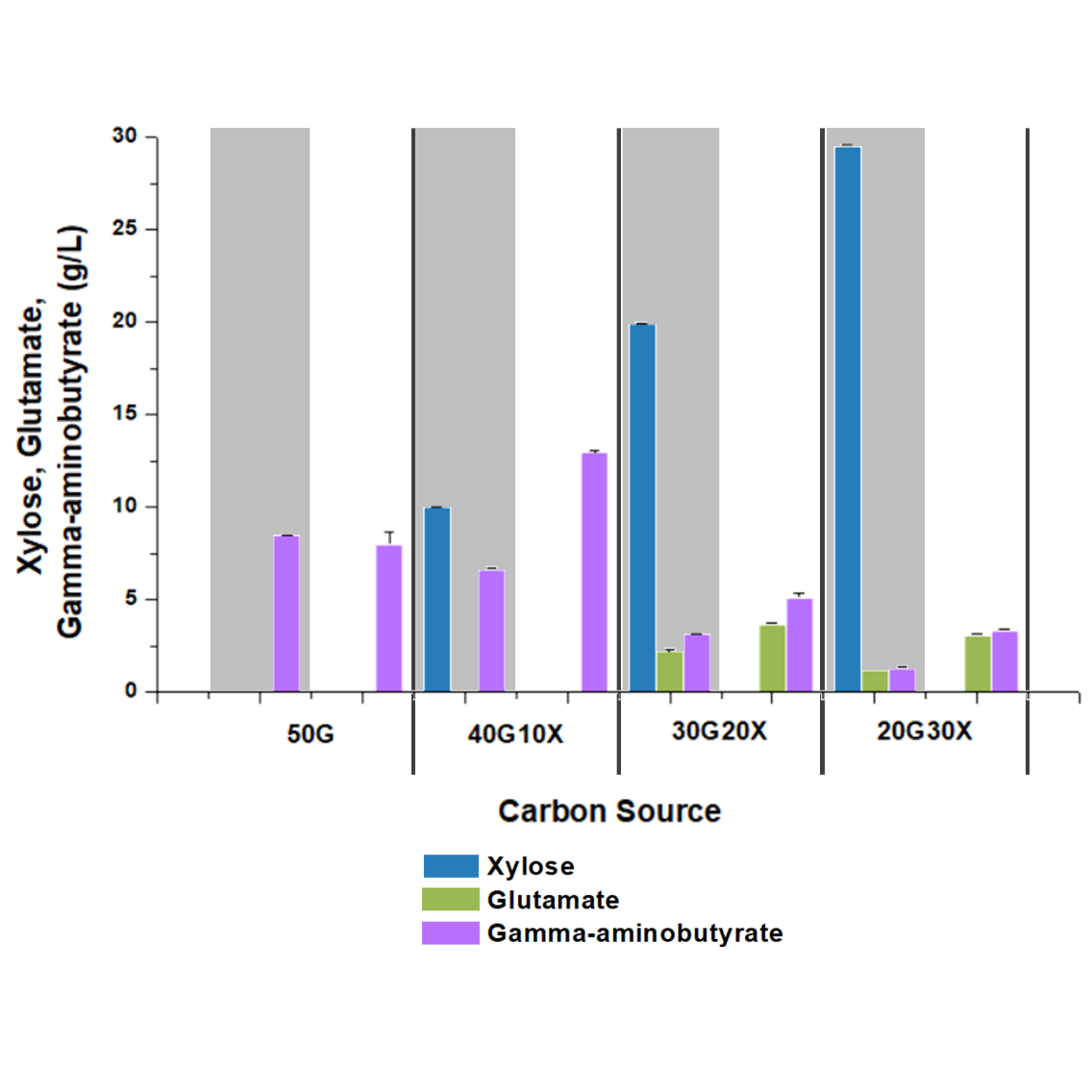


Figure S4. Gamma-aminobutyrate production by recombinant *C. glutamicum* H36GD1852 after 120 h of flask cultivation in medium containing different combinations of carbon sources (50G, 50 g/L glucose; 20G, 20 g/L glucose; 20G5X, 20 g/L glucose and 50 g/L xylose; 20G10X, 20 g/L glucose and 10 g/L xylose; 20G20X, 20 g/L glucose and 20 g/L xylose; 20G3X, 20 g/L glucose and 30 g/L xylose).


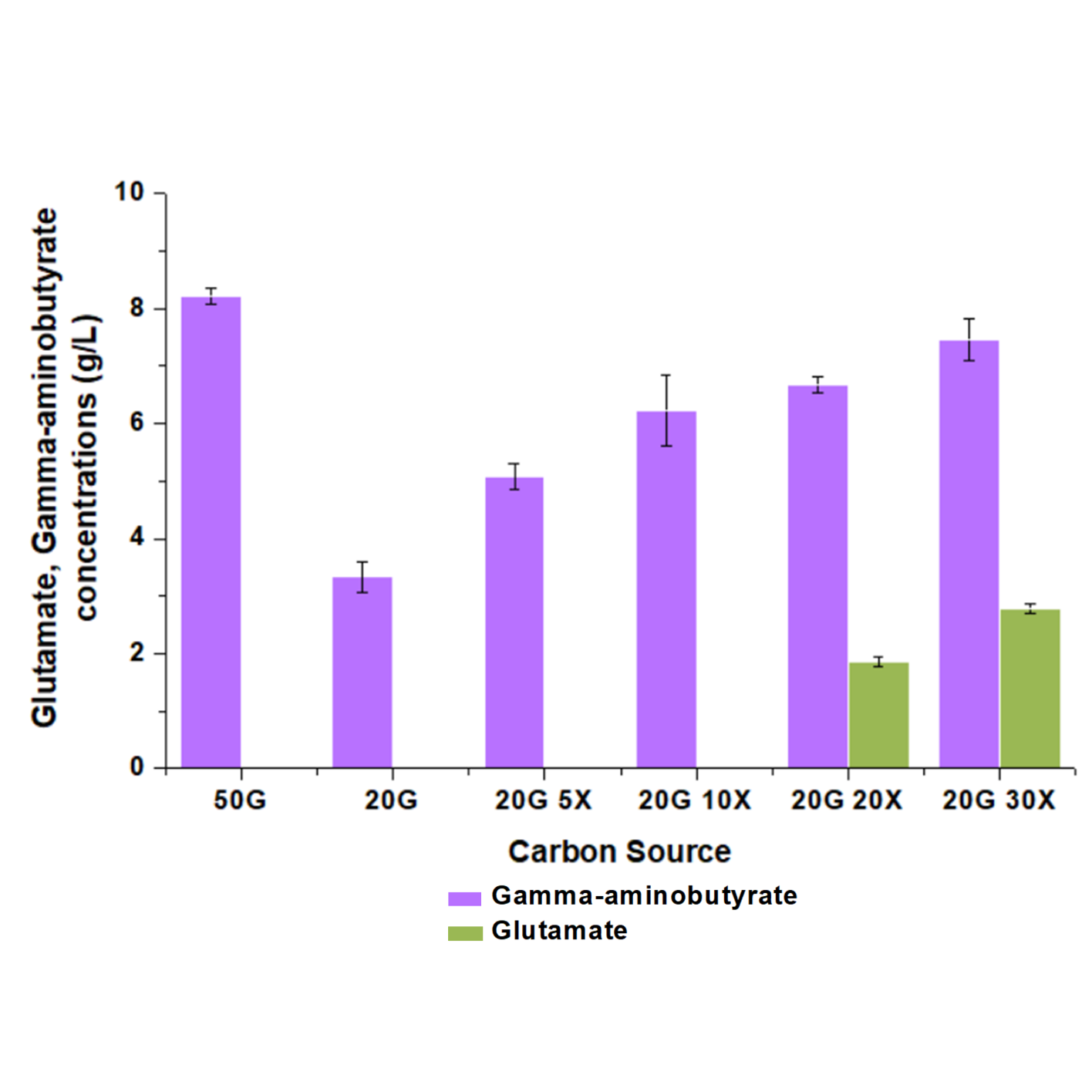


Figure S5. Gamma-aminobutyrate production and glutamate accumulation by recombinant *C. glutamicum* H36GD1852 after 120 h of flask cultivation in medium containing 30:20 glucose to xylose ratio. Additional concentrations of PLP (0.1-0.4mM) was supplemented during cultivation.


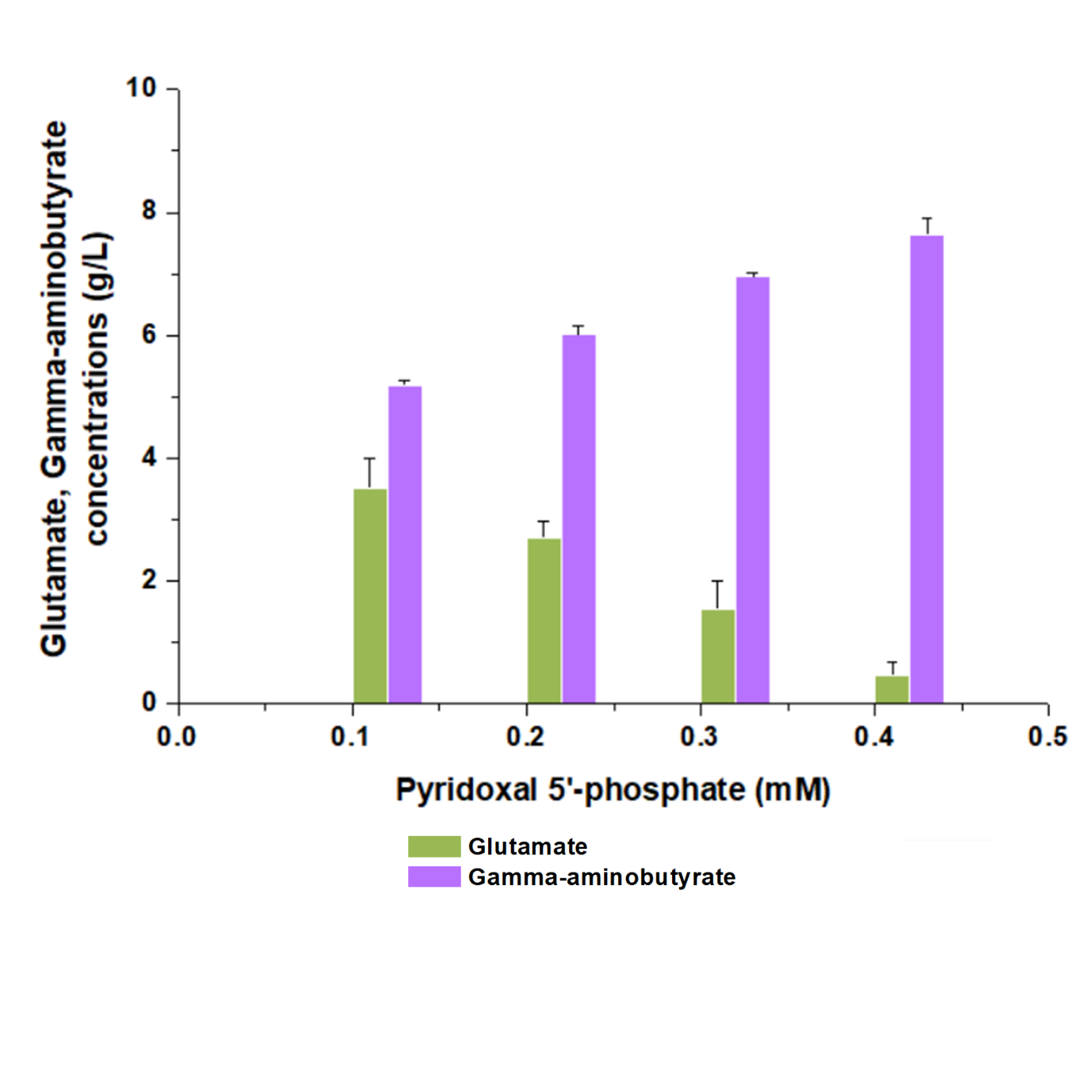

Supplement: Supplementary file 1 — Additional file 1: Table S1. Composition of empty fruit bunch (EFB) solution used as the carbon source. Figure S1. Time profiles of carbon utilization of recombinant C. glutamicum strains H36GD13032 (○, ○), H36GD1447 (△, △), and H36GD1852 (□, □) using different combinations of carbon sources. Glucose consumption is indicated as green lines and xylose is represented as blue lines (A: 50 g/L glucose, B: 40 g/L glucose, 10 g/L xylose, C: 30 g/L glucose and 20 g/L xylose, D: 20 g/L glucose and 30 g/L xylose). Figure S2. Gamma-aminobutyrate production by recombinant C. glutamicum strains H30GD13032, H30GD1447, and H30GD1852 after 120 h of flask cultivation in medium containing different combinations of carbon sources (A, 50 g/L glucose; B, 40 g/L glucose and 10 g/L xylose; C, 30 g/L glucose and 20 g/L xylose; D, 20 g/L glucose and 30 g/L xylose). Figure S3. Concentrations of xylose, glutamate and gamma-aminobutyrate after 120 h of flask cultivation using recombinant C. glutamicum H36GM1852 (gray) and C. glutamicum H36GD1852 (white). The culture medium used contained different combinations of carbon sources (50G, 50 g/L glucose; 40G10X, 40 g/L glucose and 10 g/L xylose; 30G20X, 30 g/L glucose and 20 g/L xylose; 20G30X, 20 g/L glucose and 30 g/L xylose). Figure S4. Gamma-aminobutyrate production by recombinant C. glutamicum H36GD1852 after 120 h of flask cultivation in medium containing different combinations of carbon sources (50G, 50 g/L glucose; 20G, 20 g/L glucose; 20G5X, 20 g/L glucose and 50 g/L xylose; 20G10X, 20 g/L glucose and 10 g/L xylose; 20G20X, 20 g/L glucose and 20 g/L xylose; 20G3X, 20 g/L glucose and 30 g/L xylose). Figure S5. Gamma-aminobutyrate production and glutamate accumulation by recombinant C. glutamicum H36GD1852 after 120 h of flask cultivation in medium containing 30:20 glucose to xylose ratio. Additional concentrations of PLP (0.1–0.4mM) was supplemented during cultivation. [file 12934_2018_977_MOESM1_ESM.docx]
